# Crystal structure of strontium perchlorate anhydrate, Sr(ClO_4_)_2_, from laboratory powder X-ray diffraction data

**DOI:** 10.1107/S2056989019003335

**Published:** 2019-03-15

**Authors:** Jooeun Hyoung, Hyeon Woo Lee, So Jin Kim, Hong Rim Shin, Seung-Tae Hong

**Affiliations:** a Daegu Gyeongbuk Institute of Science and Technology (DGIST), Daegu 42988, Republic of Korea

**Keywords:** crystal structure, powder X-ray diffraction, strontium perchlorate anhydrate, isotypism

## Abstract

The crystal structure of strontium perchlorate anhydrate, Sr(ClO_4_)_2_, was determined and refined from laboratory powder X-ray diffraction data. The material was obtained by dehydration of Sr(ClO_4_)_2_·3H_2_O at 523 K for two weeks. It crystallizes in the ortho­rhom­bic space group *Pbca* and is isotypic with Ca(AlD_4_)_2_ and Ca(ClO_4_)_2_. The asymmetric unit contains one Sr, two Cl and eight O sites, all on general positions (Wyckoff position 8*c*). The crystal structure consists of Sr^2+^ cations and isolated ClO_4_^−^ tetra­hedra. The Sr^2+^ cation is coordinated by eight O atoms from eight ClO_4_^−^ tetra­hedra. The validity of the crystal structure model for Sr(ClO_4_)_2_ anhydrate was confirmed by the bond valence method.

## Abstract 2

The crystal structure of Sr(ClO_4_)_2_ is isotypic with its Ca homologue.

## Chemical context   

The alkaline earth metal ions (Mg^2+^, Ca^2+^, Sr^2+^ and Ba^2+^) have received attention as ion carriers for next-generation batteries (Wang *et al.*, 2013 ▸), and their perchlorates are used as inorganic salts of conventional nona­queous electrolytes for electrochemical cells in Mg- and Ca-ion batteries (Whittingham *et al.*, 2018 ▸; Tchitchekova *et al.*, 2017 ▸; Padigi *et al.*, 2015 ▸). It is crucial to obtain anhydrous salts to achieve high electrochemical cell performance since hydrated salts can cause unwanted side reactions as a result of increased water content in the nona­queous electrolyte. Strontium perchlorate is highly hygroscopic and exists in several hydrated forms. So far, Sr(ClO_4_)_2_·3H_2_O, Sr(ClO_4_)_2_·4H_2_O and Sr(ClO_4_)_2_·9H_2_O have been identified by single-crystal X-ray diffraction (Hennings *et al.*, 2014 ▸). However, the crystal structure of the anhydrous phase has not been reported to date because of the difficulty in growing single crystals. Previously, we have determined the structures of anhydrous magnesium, barium and calcium perchlorate from laboratory powder X-ray diffraction data (Lim *et al.*, 2011 ▸; Lee *et al.*, 2015 ▸, 2018 ▸). Using the same techniques for the Sr salt, we were able to determine and refine the crystal structure of strontium perchlorate anhydrate.

## Structural commentary   

The crystal structure of anhydrous strontium perchlorate, Sr(ClO_4_)_2_, is isotypic with Ca(AlD_4_)_2_ (Sato *et al.*, 2009 ▸) and Ca(ClO_4_)_2_ (Lee *et al.*, 2018 ▸). Compared with Ca(ClO_4_)_2_, the unit-cell parameters *a*, *b* and *c* of Sr(ClO_4_)_2_ are increased by 3.0, 2.9 and 3.4%, respectively, because Sr^2+^ (1.26 Å for eight-coordination) has a larger ionic radius than Ca^2+^ (1.12 Å for eight-coordination; Shannon, 1976 ▸).

**Figure 1:**
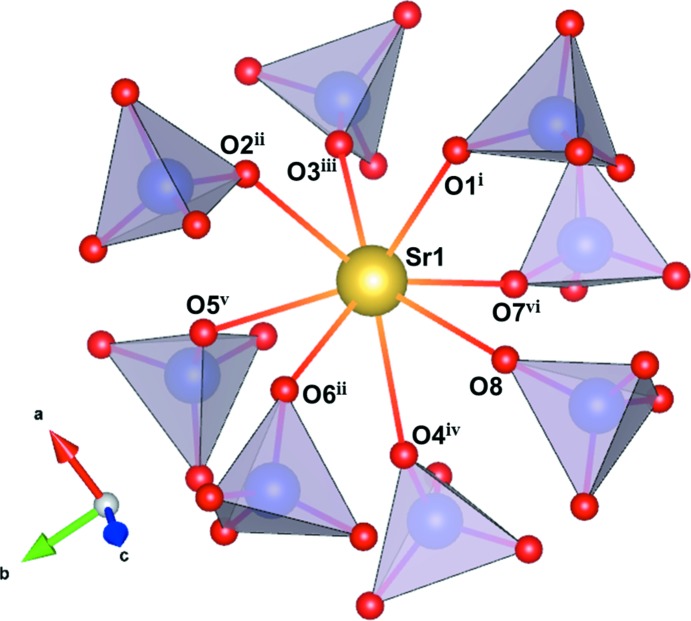
The local environment of the Sr^2+^ cation (yellow sphere) surrounded by ClO_4_^−^ tetra­hedra (purple). Symmetry codes refer to Table 1 ▸.

**Figure 2:**
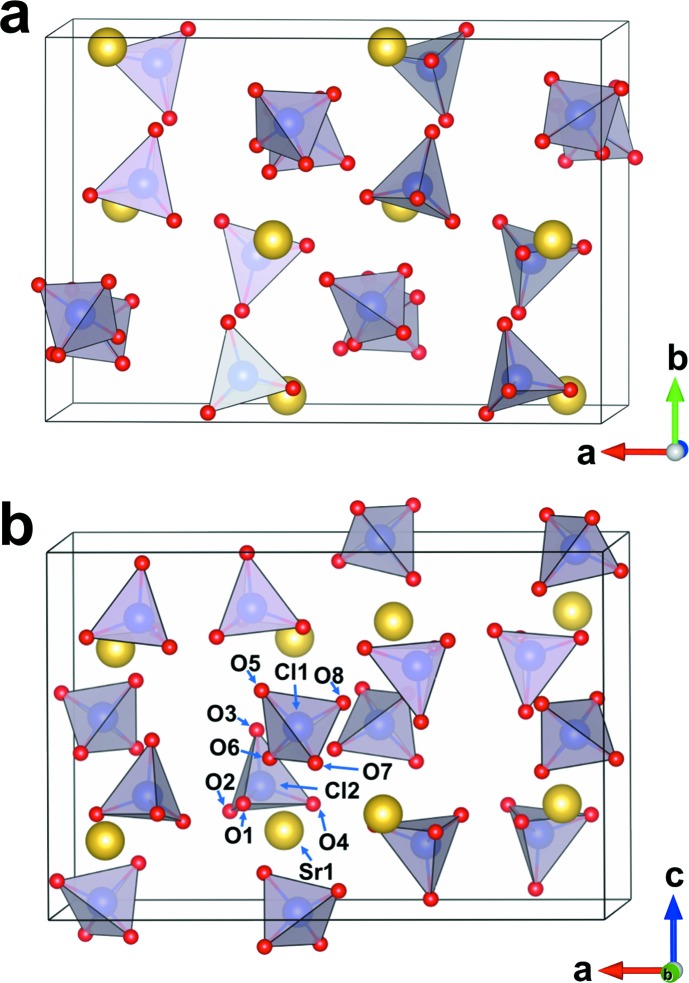
The crystal structure of Sr(ClO_4_)_2_ in two different viewing directions, *i.e.* approximately along (*a*) [001] and (*b*) [010]. Sr^2+^ cations are yellow and ClO_4_ tetra­hedra are purple.

There are one Sr, two Cl and eight O sites in the asymmetric unit, all on general positions 8*c*. The crystal structure (Fig. 1 ▸) is composed of Sr^2+^ cations and isolated ClO_4_^−^ tetra­hedra. The isolated ClO_4_^−^ tetra­hedra are slightly distorted and exhibit a range of 105.4 (7)–113.5 (7)° for the O—Cl—O angles. The local environment around the Sr^2+^ cation is presented in Fig. 2 ▸. It is coordinated by eight O atoms from eight ClO_4_^−^ tetra­hedra, with an average Sr—O distance of 2.582 Å (Table 1 ▸). The latter is inter­mediate between those of Ca—O (2.476 Å; Lee *et al.*, 2018 ▸) and Ba—O (2.989 Å; Lee *et al.*, 2015 ▸) polyhedra, and in good agreement with the sum of the ionic radii of the respective alkaline earth metal and oxygen ions (Shannon, 1976 ▸).

Empirical bond valence sums (BVSs) can be used to check structure models (Brown, 2002 ▸). In this regard, the BVSs for the ions in the crystal structure of Sr(ClO_4_)_2_ were calculated with the program *Valence* (Brown & Altermatt, 1985 ▸; Brese & O’Keeffe, 1991 ▸; Hormillosa *et al.*, 1993 ▸). The expected charges of the ions match well with the obtained BVS values (given in valence units), thus confirming the validity of the crystal structure: Sr 2.18, Cl1 6.99, Cl2 6.96, O1 1.91, O2 2.08, O3 2.06, O4 2.03, O5 1.96, O6 2.02, O7 2.03 and O8 2.04.

## Synthesis and crystallization   

Anhydrous strontium perchlorate was obtained by dehydration of Sr(ClO_4_)_2_·3H_2_O (98%, Alfa Aesar). The hydrated Sr(ClO_4_)_2_ powder was ground thoroughly in an agate mortar and added to a glass bottle. The bottle was placed in an oven at 523 K for two weeks under atmospheric conditions and then transferred to a glove-box under an argon atmosphere. For the powder X-ray diffraction measurements, anhydrous Sr(ClO_4_)_2_ was again ground in an agate mortar and placed in a tightly sealed dome-type X-ray sample holder commercially available from Bruker. The dome was double-sealed with vacuum grease to prevent hydration during measurement.

## Refinement details   

**Figure 3:**
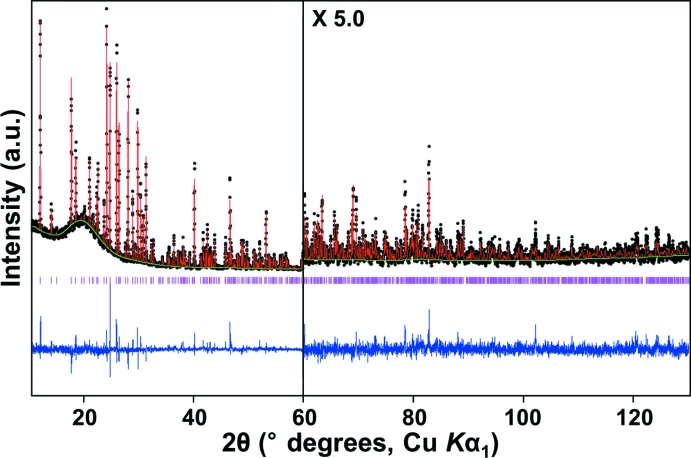
PXRD Rietveld refinement profiles for anhydrous Sr(ClO_4_)_2_ measured at ambient temperature. Black dots mark experimental data, the solid red line represents the calculated profile and the solid green line is the background. The bottom trace presents the difference curve (blue) and the ticks denote the expected Bragg reflection positions (magenta).

Details of the crystal data collection and structure refinement are summarized in Table 2 ▸. Powder X-ray diffraction (PXRD) data for anhydrous Sr(ClO_4_)_2_ were collected from a Bragg–Brentano diffractometer (PANalytical Empyrean) using Cu *K*α_1_ radiation, a focusing primary Ge(111) monochromator (λ = 1.5406 Å) and a position-sensitive PIXcel 3D 2×2 detector. The angular range was 10 ≤ 2θ ≤ 130°, with a step of 0.0131° and a total measurement time of 8 h at room temperature. The PXRD pattern was indexed using the *TREOR90* algorithm (Werner, 1990 ▸) run in *CRYSFIRE* (Shirley, 2002 ▸) through the positions of 23 reflections, resulting in an ortho­rhom­bic unit cell. Systematic reflection conditions suggested the space group *Pbca*. The crystal structure was determined by a combination of the powder profile refinement program *GSAS* (Larson & Von Dreele, 2000 ▸) and the single-crystal structure refinement program *CRYSTALS* (Betteridge *et al.*, 2003 ▸). For a three-dimensional view of the Fourier electron-density maps, *MCE* was applied (Rohlícek & Husák, 2007 ▸). Initially, a structural model with only one dummy atom at an arbitrary position in the unit cell was used. Structure factors were extracted from the powder data and then direct methods were applied to calculate the initial solution of the crystal structure using *SHELXS97* (Sheldrick, 2008 ▸) run in *CRYSTALS*, which yielded the Sr site as a starting atomic position. The initial dummy atom model was then replaced with the partial model, and this data was adopted for a Le Bail fit in *GSAS*. Improved structure factors were then extracted, which were used for the refinement in *CRYSTALS*. Such processes were iterated until a complete and satisfactory structural model was obtained. Finally, Rietveld refinement in *GSAS* was employed to complete the structure model, resulting in reasonable isotropic displacement parameters and agreement indices. For the final Rietveld refinement with *GSAS*, the Sr—O and Cl—O bond lengths were restrained with a tolerance value of 2% with respect to the distances determined from *CRYSTALS*, which matched reasonably well with the radii sums of Shannon (1976 ▸). The final Rietveld plot is displayed in Fig. 3 ▸.

## Supplementary Material

Crystal structure: contains datablock(s) I. DOI: 10.1107/S2056989019003335/wm5484sup1.cif

CCDC reference: 1901870

Additional supporting information:  crystallographic information; 3D view; checkCIF report

## Figures and Tables

**Table 1:** Selected bond lengths (Å)

Sr1—O1^i^	2.512 (5)	Cl1—O5	1.436 (5)
Sr1—O2^ii^	2.591 (5)	Cl1—O6	1.436 (5)
Sr1—O3^iii^	2.546 (5)	Cl1—O7	1.426 (5)
Sr1—O4^iv^	2.622 (5)	Cl1—O8	1.423 (5)
Sr1—O5^v^	2.650 (5)	Cl2—O1	1.469 (5)
Sr1—O6^ii^	2.540 (5)	Cl2—O2	1.414 (5)
Sr1—O7^vi^	2.590 (5)	Cl2—O3	1.425 (5)
Sr1—O8	2.604 (8)	Cl2—O4	1.422 (5)

Symmetry codes: (i) 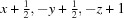; (ii) 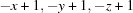; (iii) 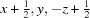; (iv) 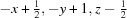; (v) 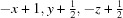; (vi) 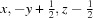.

**Table 2:** Experimental details

Crystal data	
Chemical formula	Sr(ClO_4_)_2_
*M*_r_	286.52
Crystal system, space group	Orthorhombic, *P* *b* *c* *a*
Temperature (K)	298
*a*, *b*, *c* (Å)	14.18206 (10), 9.78934 (11), 9.37624 (10)
*V* (Å^3^)	1301.73 (2)
*Z*	8
Radiation type	Cu *K*α_1_, λ = 1.5405 Å
Specimen shape, size (mm)	Flat sheet, 24.9 × 24.9

Data collection	
Diffractometer	PANalytical Empyrean
Specimen mounting	Packed powder
Data collection mode	Reflection
Scan method	Step
2θ values (°)	2θ_min_ = 10.009, 2θ_max_ = 129.991, 2θ_step_ = 0.013

Refinement	
*R* factors and goodness of fit	*R*_p_ = 0.086, *R*_wp_ = 0.125, *R*_exp_ = 0.096, *R*(*F* ^2^) = 0.14871, χ^2^ = 1.716
No. of parameters	40
No. of restraints	23

Computer programs: *X’Pert Data Collector* (PANalytical, 2011 ▸), *GSAS* (Larson & Von Dreele, 2000 ▸), *X’Pert HighScore Plus* (PANalytical, 2011 ▸), *SHELXS97* (Sheldrick, 2008 ▸), *CRYSTALS* (Betteridge *et al.*, 2003 ▸) and *VESTA* (Momma & Izumi, 2011 ▸).

## supplementary crystallographic information

### Crystal data

**Table:**

Sr(ClO_4_)_2_	*Z* = 8
*M**_r_* = 286.52	*F*(000) = 1088.0
Orthorhombic, *P**b**c**a*	*D*_x_ = 2.925 Mg m^−^^3^
Hall symbol: -P_2ac_2ab	Cu *K*α_1_ radiation, λ = 1.5405 Å
*a* = 14.18206 (10) Å	*T* = 298 K
*b* = 9.78934 (11) Å	white
*c* = 9.37624 (10) Å	flat_sheet, 24.9 × 24.9 mm
*V* = 1301.73 (2) Å^3^	Specimen preparation: Prepared at 298 K

### Data collection

**Table:**

PANalytical Empyrean diffractometer	Data collection mode: reflection
Radiation source: sealed X-ray tube, PANalytical Cu Ceramic X-ray tube	Scan method: step
Specimen mounting: packed powder	2θ_min_ = 10.009°, 2θ_max_ = 129.991°, 2θ_step_ = 0.013°

### Refinement

**Table:**

Least-squares matrix: full	Profile function: CW Profile function number 4 with 18 terms Pseudovoigt profile coefficients as parameterized in P. Thompson, D.E. Cox & J.B. Hastings (1987). J. Appl. Cryst.,20,79-83. Asymmetry correction of L.W. Finger, D.E. Cox & A. P. Jephcoat (1994). J. Appl. Cryst.,27,892-900. Microstrain broadening by P.W. Stephens, (1999). J. Appl. Cryst.,32,281-289. #1(GU) = 0.000 #2(GV) = 0.000 #3(GW) = 0.000 #4(GP) = 9.252 #5(LX) = 0.900 #6(ptec) = 0.00 #7(trns) = 0.00 #8(shft) = -3.8100 #9(sfec) = 0.00 #10(S/L) = 0.0208 #11(H/L) = 0.0005 #12(eta) = 0.7500 #13(S400 ) = 0.0E+00 #14(S040 ) = 7.8E-04 #15(S004 ) = 1.5E-04 #16(S220 ) = 3.7E-04 #17(S202 ) = 6.1E-04 #18(S022 ) = -1.1E-03 Peak tails are ignored where the intensity is below 0.0010 times the peak Aniso. broadening axis 0.0 0.0 1.0
*R*_p_ = 0.086	40 parameters
*R*_wp_ = 0.125	23 restraints
*R*_exp_ = 0.096	(Δ/σ)_max_ = 0.05
*R*(*F*^2^) = 0.14871	Background function: GSAS Background function number 1 with 34 terms. Shifted Chebyshev function of 1st kind 1: 118.082 2: -166.900 3: 123.865 4: -59.7925 5: 10.6865 6: 21.3336 7: -31.0975 8: 23.2200 9: -6.96687 10: -9.51726 11: 20.8794 12: -23.8022 13: 18.4347 14: -9.14997 15: -1.10995 16: 9.35323 17: -13.3633 18: 13.2873 19: -9.61569 20: 4.08246 21: 1.61524 22: -5.79316 23: 6.77390 24: -5.01271 25: 2.27833 26: 0.646733 27: -2.78842 28: 3.78393 29: -3.23100 30: 2.18997 31: -0.908158 32: -0.401332 33: 0.778547 34: -0.792308
9139 data points	Preferred orientation correction: March-Dollase AXIS 1 Ratio= 0.79858 h= 1.000 k= 0.000 l= 0.000 Prefered orientation correction range: Min= 0.71363, Max= 1.96360

### Fractional atomic coordinates and isotropic or equivalent isotropic displacement parameters (Å^2^)

**Table:**

	*x*	*y*	*z*	*U*_iso_*/*U*_eq_	
Sr1	0.60125 (9)	0.46444 (17)	0.2136 (2)	0.0206 (3)*	
Cl1	0.4370 (3)	0.2794 (4)	0.4905 (4)	0.0254 (3)*	
Cl2	0.1592 (2)	0.3965 (4)	0.6866 (4)	0.0275 (3)*	
O1	0.1833 (5)	0.2549 (6)	0.7237 (14)	0.0271 (14)*	
O2	0.2200 (4)	0.4878 (11)	0.7587 (12)	0.0335 (14)*	
O3	0.1636 (7)	0.4175 (13)	0.5363 (6)	0.0364 (14)*	
O4	0.0665 (4)	0.4250 (10)	0.7362 (11)	0.0225 (14)*	
O5	0.3759 (6)	0.2145 (7)	0.3888 (11)	0.0356 (14)*	
O6	0.3829 (6)	0.3692 (11)	0.5799 (10)	0.0361 (14)*	
O7	0.4728 (6)	0.1764 (11)	0.5835 (12)	0.0315 (14)*	
O8	0.5132 (6)	0.3514 (12)	0.4267 (12)	0.0305 (14)*	

### Geometric parameters (Å, º)

**Table:**

Sr1—Cl1	3.931 (4)	Cl2—Sr1^ix^	3.945 (3)
Sr1—Cl1^i^	3.937 (4)	Cl2—Sr1^ii^	3.778 (3)
Sr1—Cl1^ii^	3.779 (4)	Cl2—Sr1^x^	3.746 (4)
Sr1—Cl1^iii^	3.669 (4)	Cl2—Sr1^xi^	3.898 (4)
Sr1—Cl2^iv^	3.945 (3)	Cl2—O1	1.469 (5)
Sr1—Cl2^ii^	3.778 (3)	Cl2—O2	1.414 (5)
Sr1—Cl2^v^	3.746 (4)	Cl2—O3	1.425 (5)
Sr1—Cl2^vi^	3.898 (4)	Cl2—O4	1.422 (5)
Sr1—O1^v^	2.512 (5)	O1—Sr1^x^	2.512 (5)
Sr1—O2^ii^	2.591 (5)	O1—Cl2	1.469 (5)
Sr1—O3^vi^	2.546 (5)	O2—Sr1^ii^	2.591 (5)
Sr1—O4^iv^	2.622 (5)	O2—Cl2	1.414 (5)
Sr1—O5^iii^	2.650 (5)	O3—Sr1^xi^	2.546 (5)
Sr1—O6^ii^	2.540 (5)	O3—Cl2	1.425 (5)
Sr1—O7^i^	2.590 (5)	O4—Sr1^ix^	2.622 (5)
Sr1—O8	2.604 (8)	O4—Cl2	1.422 (5)
Cl1—Sr1	3.931 (4)	O5—Sr1^viii^	2.650 (5)
Cl1—Sr1^vii^	3.937 (4)	O5—Cl1	1.436 (5)
Cl1—Sr1^ii^	3.779 (4)	O6—Sr1^ii^	2.540 (5)
Cl1—Sr1^viii^	3.669 (4)	O6—Cl1	1.436 (5)
Cl1—O5	1.436 (5)	O7—Sr1^vii^	2.590 (5)
Cl1—O6	1.436 (5)	O7—Cl1	1.426 (5)
Cl1—O7	1.426 (5)	O8—Sr1	2.604 (8)
Cl1—O8	1.423 (5)	O8—Cl1	1.423 (5)
			
O1^v^—Sr1—O2^ii^	71.2 (3)	O5^iii^—Sr1—O8	136.8 (4)
O1^v^—Sr1—O3^vi^	84.3 (4)	O6^ii^—Sr1—O7^i^	139.7 (3)
O1^v^—Sr1—O4^iv^	138.9 (3)	O6^ii^—Sr1—O8	74.3 (4)
O1^v^—Sr1—O5^iii^	144.9 (3)	O7^i^—Sr1—O8	78.3 (4)
O1^v^—Sr1—O6^ii^	109.2 (4)	O5—Cl1—O6	109.7 (5)
O1^v^—Sr1—O7^i^	88.9 (4)	O5—Cl1—O7	108.0 (7)
O1^v^—Sr1—O8	71.3 (4)	O5—Cl1—O8	113.5 (7)
O2^ii^—Sr1—O3^vi^	77.6 (3)	O6—Cl1—O7	105.4 (7)
O2^ii^—Sr1—O4^iv^	143.6 (3)	O6—Cl1—O8	110.3 (7)
O2^ii^—Sr1—O5^iii^	75.5 (3)	O7—Cl1—O8	109.7 (7)
O2^ii^—Sr1—O6^ii^	73.8 (3)	O1—Cl2—O2	110.0 (7)
O2^ii^—Sr1—O7^i^	146.3 (3)	O1—Cl2—O3	111.1 (7)
O2^ii^—Sr1—O8	118.0 (4)	O1—Cl2—O4	108.8 (6)
O3^vi^—Sr1—O4^iv^	117.7 (3)	O2—Cl2—O3	110.7 (8)
O3^vi^—Sr1—O5^iii^	77.9 (3)	O2—Cl2—O4	106.4 (6)
O3^vi^—Sr1—O6^ii^	141.9 (4)	O3—Cl2—O4	109.6 (6)
O3^vi^—Sr1—O7^i^	73.4 (3)	Sr1^x^—O1—Cl2	139.0 (5)
O3^vi^—Sr1—O8	142.8 (4)	Sr1^ii^—O2—Cl2	139.4 (5)
O4^iv^—Sr1—O5^iii^	76.1 (3)	Sr1^xi^—O3—Cl2	157.0 (6)
O4^iv^—Sr1—O6^ii^	75.8 (3)	Sr1^ix^—O4—Cl2	153.3 (6)
O4^iv^—Sr1—O7^i^	67.6 (3)	Sr1^viii^—O5—Cl1	125.1 (4)
O4^iv^—Sr1—O8	71.2 (4)	Sr1^ii^—O6—Cl1	142.2 (5)
O5^iii^—Sr1—O6^ii^	70.9 (4)	Sr1^vii^—O7—Cl1	156.2 (7)
O5^iii^—Sr1—O7^i^	114.04 (3)	Sr1—O8—Cl1	153.9 (7)

Symmetry codes: (i) *x*, −*y*+1/2, *z*−1/2; (ii) −*x*+1, −*y*+1, −*z*+1; (iii) −*x*+1, *y*+1/2, −*z*+1/2; (iv) −*x*+1/2, −*y*+1, *z*−1/2; (v) *x*+1/2, −*y*+1/2, −*z*+1; (vi) *x*+1/2, *y*, −*z*+1/2; (vii) *x*, −*y*+1/2, *z*+1/2; (viii) −*x*+1, *y*−1/2, −*z*+1/2; (ix) −*x*+1/2, −*y*+1, *z*+1/2; (x) *x*−1/2, −*y*+1/2, −*z*+1; (xi) *x*−1/2, *y*, −*z*+1/2.
